# Trapped in a Gray Zone of Isolation: A Qualitative Study on the Social Withdrawal and Isolation Experiences of Youth NEETs in South Korea

**DOI:** 10.3390/bs16101906

**Published:** 2026-10-15

**Authors:** Jinjoo Hong

**Affiliations:** Department of Social Welfare, Hannam University, Daejeon 34430, Republic of Korea; pearl0606@hnu.ac.kr; Tel.: +82-42-629-7651

**Keywords:** Youth NEET, social exclusion, social isolation, social withdrawal, qualitative case study

## Abstract

Young people not in employment, education, or training (hereafter, Youth NEETs) have been examined in South Korea primarily through quantitative indicators, leaving the lived experience of their social isolation and withdrawal largely unexplored. This study explored how Korean Youth NEETs experience isolation and withdrawal, and how these states are formed and sustained within their life contexts. A qualitative multiple-case study was conducted with eight non-job-seeking Youth NEETs aged 19–34, recruited purposively in Seoul. Data were collected through repeated in-depth interviews and were analyzed through within-case analysis followed by Stake’s cross-case analysis. Five main categories, 11 subcategories, and 33 meaning units emerged: the shadow of marginalization; being pushed out into the world unprepared; being treated as expendable in an unstable labor market; failing on the high threshold of employment; and being trapped in a gray zone of isolation. Participants experienced cumulative and multidimensional social exclusion across family, school, the labor market, and social relationships, which manifested psycho-emotionally as learned helplessness, self-stigma, avoidant coping, and depressive distress. The findings suggest that NEET status should be reframed as a problem of multidimensional social exclusion rather than individual failure, and that integrated, stage-based psycho-emotional support is needed alongside employment-centered policies.

## 1. Introduction

The school-to-work transition in young adulthood is a critical developmental task in the life course. Many young people today, however, no longer experience the stable transitions of the past; amid changing social environments, they undergo an ‘emerging adulthood’ marked by delay and postponement of the transition process (Arnett, 2007). Alongside discussions of this growing instability, attention has increasingly turned to NEETs (Not in Employment, Education, or Training)—young people who have failed in, or deferred, the transition from school to the labor market and belong to none of the domains of education, employment, or training (Byun, 2025; M. Park et al., 2020). NEET is a relatively new indicator that captures the vulnerability of young people by identifying the economically inactive population-such as discouraged job seekers-whom conventional labor indicators, which classify people only as employed or unemployed, have failed to detect (Mascherini et al., 2012; Roberts, 2011). The NEET phenomenon is not confined to any single society but is observed worldwide. In the member countries of the Organization for Economic Co-operation and Development (OECD) and in the European Union (EU), the NEET rate is managed as a key indicator of youth vulnerability, and prolonged NEET status has been reported to adversely affect subsequent employment, income, psychosocial well-being, and health, while entailing substantial social costs (Lőrinc et al., 2020; Mascherini et al., 2012).

Youth NEETs occupy a position ‘outside society’, in a ‘gray zone’ (Mascherini et al., 2012) beyond formal social systems, and this very positioning exposes them to social exclusion and to withdrawal within relationships. When such experiences of exclusion and withdrawal become prolonged or accumulate across multiple dimensions, they carry the risk of deepening into a state of social isolation and withdrawal in which young people cut themselves off from society. At this juncture, the NEET problem needs to be re-examined not merely as a labor issue of employment, but as a crisis of social relationships and mental health encompassing social exclusion, withdrawal, isolation, and seclusion.

As of 2022, the NEET rate among Korean youth was 20.1% by the OECD definition, and an estimated 17.1% when Korea’s social context (participation in private academies and non-formal vocational training) is taken into account—still 4.5 percentage points above the OECD average of 12.6% (K. Kim, 2024). Against this backdrop of expanding social concern over young people—including the long-term unemployed, discouraged job seekers, and socially isolated and withdrawn youth—Youth NEETs have come to be labeled as a representative and symbolic group harboring social risk (C. H. Lee, 2018). They are viewed from multiple, shifting perspectives; sometimes as young job seekers in need of employment services, sometimes as hikikomori, sometimes as figures who embody social danger, and sometimes as a group who have simply chosen a leisurely life. Such labeling ultimately reveals a problem of social perception in which Youth NEETs are named without clear understanding, while remaining ‘yet to be explored’ (Batini et al., 2017). In other words, a more systematic and in-depth approach is needed to understand who Youth NEETs are within Korean society and what they actually experience.

Accordingly, this study conducted in-depth interviews with eight Korean Youth NEETs to explore the patterns of their withdrawal and isolation, the contexts in which these states are formed and sustained, and the meanings the participants themselves attach to their experiences. The specific research questions were as follows. First, how do Youth NEETs experience the process of reaching isolation and withdrawal? Second, how are their daily lives and relationships constituted within the state of withdrawal and isolation? Third, what meanings do they attach to their experiences, and how do they reconstruct them? By adding the voices of the young people themselves to a phenomenon that has so far been recorded mainly through quantitative indicators, this study aims to deepen understanding of the isolation and withdrawal of Korean Youth NEETs and to derive practical implications for future practice and support policies.

### 1.1. Research on NEET Youth

Research on Youth NEETs is a significant international issue addressed across many countries. A recent global scoping review of the NEET literature (Gunnes et al., 2025), analyzing 159 studies, reported that the factors influencing NEET status are diverse—spanning individual, family, and systemic elements—and that the issues addressed range from psychological problems such as mental health difficulties and low self-esteem to social problems such as inequality, discrimination, and exclusion. In Korea, policy discussions on Youth NEETs began in the 2010s, and research has become increasingly active since the 2020s. The scholarly approaches to Youth NEETs in recent Korean research can be broadly divided into three approaches.

The first is an economic and policy-oriented approach that identifies the risk factors for becoming NEET and the structural conditions of labor market transition (Byun, 2025; M. Park et al., 2020). Indeed, the majority of Korean studies on Youth NEETs concern their current status, typologies, and influencing factors (Y. Choi, 2015; Jeon & Lee, 2024; H. E. Lee, 2024; J. Park & Han, 2024). For example, Jeon and Lee (2024) classified Youth NEETs into latent classes according to their job-seeking attitudes—‘deferred’, ‘job-seeking’, and ‘resigned’ types—and analyzed the sociodemographic, household-background, and psycho-emotional characteristics and influencing factors of each type.

The second is a clinical approach that tests the associations between employment status and mental health or quality of life (G. E. Nam et al., 2021). More recently, attempts have emerged to address NEET status at the psychological and behavioral level, for example, by analyzing the relationship between NEETs’ career expectations and social withdrawal behavior through mediation models (Xu et al., 2025). While these studies have contributed to identifying the scale of the NEET phenomenon and the correlational structures among variables, they share a common reliance on survey-based quantitative designs (H. E. Lee, 2024). An in-depth understanding, grounded in the voices of the young people themselves, of how Youth NEETs come to experience withdrawal and isolation, and how they make sense of that experience, remains lacking.

The third strand comprises qualitative studies that seek to understand the phenomenon and experiences of Youth NEETs. Research has focused on typologizing Youth NEETs or on specific intervention contexts, such as young people’s experiences of, and the effectiveness of, related programs (Bae & Kim, 2026; M. Park et al., 2020). Consequently, these studies were limited in their ability to capture the experience itself holistically—how withdrawal and isolation are formed and sustained among Youth NEETs (H. Lee et al., 2026b; Mo & Kim, 2025; Noh, 2021). To address this limitation, qualitative research on the life contexts of Youth NEETs has been undertaken. Researchers(Baldry et al., 2019; Noh, 2021; S. H. Kim, 2023) have described the NEET experience as ‘churn’, discussing situations in which advance and retreat, frustration and failure, and isolation and connection become entangled and repeated rather than following a linear transition. That is, their transition process is not a linear one; complex realities are experienced multidimensionally within context. S. H. Kim (2023), through a qualitative interpretive meta-synthesis, sought an in-depth understanding of the subjective experiential world of Korean youth NEETs, analyzing their experiences under the overarching theme of ‘the churning experiences of isolation and connection’, which comprised ‘the path of young adult development entered differently from others’, ‘insignificant daily life’, ‘fragmented shadows without a decent role’, ‘a hand trying to lift me up when I flounder without wings’ and ‘still hesitant, but carefully unfolding crumpled me’. Noh (2021) analyzed the life histories of Korean Youth NEETs and the contexts in which NEET status is formed, showing that these young people navigate their lives by drawing on turning points and adaptive strategies within the domains of their lives, and proposed institutional and policy alternatives for Korean society.

### 1.2. Psycho-Emotional Difficulties of NEET Youth

Youth NEETs are known to experience diverse difficulties and social exclusion. Research has consistently linked time spent as NEET to lasting disadvantage: these young people tend to complete less education, are more likely to be out of work later in life, and are at greater risk of mental health problems (Coles et al., 2010; Gariépy et al., 2022; Gunnes et al., 2025). What deserves attention here is the consistent finding that NEET status is not a problem of joblessness alone but is bound up with exclusion across the domains of education, work, and relationships, in the course of which young people come to experience psycho-emotional and mental health difficulties. Beyond the established link between unemployment or precarious employment and the deterioration of young people’s mental health (G. E. Nam et al., 2021; Vancea & Utzet, 2017), Youth NEETs struggle amid deepening youth unemployment and insecurity in employment and housing; they experience alienation and stigma, and undergo negative psycho-emotional states such as diminished self-esteem, loss of vitality, and depression, with a tendency to move toward avoidance, seclusion, and isolation. Research on the process of becoming NEET has discussed how young people enter NEET status through involuntary circumstances and then find it difficult to escape (Chen, 2011). Longitudinal evidence shows that being NEET in adolescence predicts poorer mental health in early adulthood, most markedly a higher risk of new-onset suicidal behavior, independent of baseline disadvantage (Gutiérrez-García et al., 2017). Discussion of the causal relationships and effects surrounding the psycho-emotional and mental health difficulties of Youth NEETs has been relatively active, particularly in international research. Numerous studies exist, including research on the links between NEET experience and mental health difficulties such as depression and substance use (Gariépy et al., 2022; Henderson et al., 2017; Lindblad et al., 2024; O’Dea et al., 2016), and a recent scoping review showing that being NEET goes hand in hand with poorer mental health, lower self-regard, and exclusion from social life (Gunnes et al., 2025). In Korea, approaches to the suicidal ideation of Youth NEETs (Ji et al., 2024; S. Kim, 2022) and to the mental health of precarious youth (Cho, 2026) have recently begun to emerge.

Meanwhile, young people’s prolonged social withdrawal and psycho-emotional difficulties—first conceptualized in Japan as ‘hikikomori’—have expanded into a transnational phenomenon observed in societies beyond Hong Kong, and Korea (Li & Wong, 2015). In particular, recent studies of Korean young adults have reported that hikikomori is significantly associated with depression and suicidal ideation (Baek & Yoon, 2025; S. M. Park et al., 2025). Related work has linked social isolation to adverse childhood experiences (H. Choi & Hwang, 2023) and to unemployment, depression, relative deprivation, and low self-esteem (S.-B. Lee et al., 2022), and has shown that discrepancies between online and offline relational experiences strengthen the pathway from psychological vulnerability through depression to hikikomori (C. M. Lee & La, 2026). In the Korean context, intense competition, prolonged and unstable transitions from education to work, and insecure employment and housing are deepening young people’s mental health difficulties (Cho, 2026; C. M. Lee & La, 2026; S.-B. Lee et al., 2022);; for vulnerable groups such as Youth NEETs in particular, these difficulties are further compounded by the social exclusion they experience. Taken together, this body of research supports the need for a psycho-emotional approach to Youth NEETs

In sum, isolation and withdrawal among Korean young people, including Youth NEETs, have been reported in international journals mainly through quantitative studies based on surveys and epidemiological data, and qualitative research exploring, from the perspective of the young people themselves, how these experiences are formed and sustained remains rare. Even qualitative studies of Youth NEETs have largely been confined to documenting and evaluating participation in support projects and programs, rather than examining the experience of isolation and withdrawal itself (Bae & Kim, 2026; Kwon & Lee, 2024; H. Lee et al., 2026a; E. Park, 2022). An understanding, from young people’s own perspective, of how isolation and withdrawal are formed and sustained within the contexts of their lives therefore remains lacking, and the present study addresses this gap.

### 1.3. Conceptual Framework of the Present Study

This study conceptualizes the isolation and withdrawal of Youth NEETs as the outcome of an interaction between structural exclusion and its psycho-emotional internalization that unfolds across three levels. At the macro level, the developmental context of emerging adulthood (Arnett, 2007) intersects with a segmented and precarious labor market: transitions are prolonged and non-linear, and young people with limited family and educational resources face a higher risk of NEET status and unstable employment (Mascherini et al., 2012). At the mezzo level, social exclusion is understood not as a single event but as a dynamic and cumulative process in which deprivation in one domain—family, school, the labor market, or social relationships— can reinforce deprivation in the others (Levitas et al., 2007). At the micro level, this study draws on three psychological constructs to theorize how cumulative exclusion is internalized: repeated experiences of failure perceived as uncontrollable foster learned helplessness (Seligman, 1975); the internalization of negative societal labels directed at NEETs produces self-stigma (Corrigan & Watson, 2002); and, when situational demands are appraised as exceeding available resources (Lazarus & Folkman, 1984), avoidant coping is adopted, expressed behaviorally as withdrawal from relationships and retreat into home and online spaces. These processes form a feedback loop: withdrawal further severs social ties and opportunities, deepening exclusion, and may culminate in prolonged seclusion of the kind conceptualized as hikikomori (Li & Wong, 2015). Within this framework, social withdrawal is understood as a behavioral retreat from relationships and participation, social isolation as the objective lack of supportive ties, and seclusion (hikikomori) as an extreme, home-confined form of withdrawal; the “gray zone” examined here designates the liminal state in which Youth NEETs hover among these conditions. The three research questions operationalize this framework by asking how exclusion accumulates into withdrawal (RQ1), how daily life and relationships are constituted within it (RQ2), and how participants make meaning of the experience (RQ3).

## 2. Materials and Methods

### 2.1. Participants

The participants were defined—adapting the category of restricted NEET (Serracant, 2014)—as ‘non-disabled young people aged 19 to 34 who, during the preceding year, had not been enrolled in school, had not been employed, had not attended private academies or vocational training institutions for the purpose of further education or employment, and had not been actively seeking employment’. The study focused on ‘non-job-seeking NEETs’, who have been relatively marginalized in existing youth support policies. On the assumption that job-seeking NEETs are likely to be connected to youth unemployment programs, the study restricted its focus to non-job-seeking NEETs who experience exclusion from institutional connections (J. Nam, 2011). Homemakers, such as women whose careers had been interrupted, and young people with disabilities were excluded, as these are groups for which separate discourses of exclusion have formed and their inclusion could confound understanding of the characteristics of the school-to-work transition. Because young people with long NEET experience may have no choice but to take intermittent day labor or short-term work to subsist, such work was not treated as employment. The 19–34 age range corresponds to the legal definition of “youth” in the Framework Act on Youth (2020), on which Korean youth-policy statistics, including NEET estimates, are compiled; the exclusion of young people with disabilities refers to registered disability status under the Korean disability registration system.

The one-year criterion was set to ensure that participants had experienced NEET status long enough for its characteristic features to be evident, given that young people frequently move in and out of NEET status the characteristic experiences of being NEET would be clearly manifested, given the reality of frequent entry into and exit from NEET status (R. Lee et al., 2010). Other sociodemographic characteristics such as gender, educational attainment, and income were not considered in the selection criteria. Participants were recruited through purposive sampling, guided by the criteria of appropriateness (selecting participants able to provide rich information relevant to the research purpose) and sufficiency (collecting data until saturation) (Shin et al., 2004); the recruitment pathway is described below. The general characteristics of the eight final participants are presented in Table 1. Notably, five of the participants were experiencing mental health difficulties involving depression, three were experiencing difficulties with interpersonal avoidance, and some had histories of treatment for conditions such as ADHD and game addiction. All information on psycho-emotional and relational difficulties was based on participants’ own accounts during the interviews; no information on participants’ psychological or health conditions was obtained from organizational staff, and no clinical screening, assessment, or diagnosis was conducted by the researcher.

The researcher contacted social welfare and youth-support organizations in Seoul and Gyeonggi Province that operate programs for adolescents and young adults, explained the purpose of the study, and requested that they publicize the study and recommend candidates meeting the eligibility criteria. The role of organizational staff was limited to identifying potential participants and confirming basic eligibility—current NEET status, absence of job-seeking activity, and connection to the organization; no information provided by staff was used as research data or in the interpretation of findings. Identifying eligible candidates proved difficult, as most organizations either applied broader definitions of NEET or served low-income adolescents preventively—an experience that itself underscored the need for conceptual consensus on Youth NEETs in the Korean context. Ultimately, nine candidates were recommended through six organizations: three community welfare centers, two social economy organizations, and one non-profit corporation, all in Seoul. All nine were interviewed. One was excluded during the interview process when it emerged that a course the participant was attending was part of the government Employment Success Package, which violated the eligibility criterion of non-participation in employment-purpose training; the final sample therefore comprised eight participants. Although the initial plan prioritized candidates able to communicate their experiences verbally without difficulty, one recommended candidate who experienced severe difficulty with face-to-face communication was retained, as the case carried particular significance as a single-person-household Youth NEET; interviews with this participant were adapted accordingly (see Section 2.2). All participants received a cash honorarium upon completion of their third interview.

### 2.2. Data Collection

Data were collected through individual in-depth interviews from November 2018 to December 2019. To secure sufficient data for each case, one-on-one interviews were adopted, with a target of three or more sessions per participant to achieve depth within each case. As a result, seven participants each completed three face-to-face in-depth interviews, for a total of 21 sessions; one participant, who had difficulty with face-to-face communication owing to severe social withdrawal, completed three e-mail interviews following an initial meeting held to explain the study, obtain written consent, and establish rapport— yielding a corpus of 22 face-to-face sessions and three e-mail interviews in total. All face-to-face interviews were conducted in Korean, audio-recorded with participants’ consent, and transcribed verbatim; each session comprised approximately 60 min of substantive interviewing, excluding time for greetings and rapport-building. The e-mail interviews were conducted with the participant’s written informed consent, in a reply-based format in which the participant responded from the participant’s own account to questions sent by the researcher; the correspondence was converted into anonymized transcripts and retained under the same restricted-access conditions as the audio recordings, in accordance with the IRB-approved data-management plan. Interviews were conducted at times and places where participants felt comfortable, mainly in the counseling rooms of the recommending organizations or, at some participants’ request, in quiet local cafes, where seating was chosen to prevent conversations from being overheard. The semi-structured interview guide was composed of items covering the major domains of social exclusion identified in prior research and items for understanding participants’ daily lives and experiences, including sociodemographic characteristics; family relationships; economic circumstances; daily life and health; experiences of school; work, job seeking, and relationships; social activities; welfare experiences, and perceptions of NEET status and the future. The guide (ver. 1) was reviewed as part of the IRB-approved protocol, and its main domains and representative questions are presented in Appendix A. Notably, the social-exclusion items served solely as the researcher’s sensitizing reference for probing and contextual verification and were never posed directly to participants, whose accounts were elicited through open questions about school, work, job seeking, daily life, and perceptions of NEET status. During the interviews, topics were explored in greater depth and adjusted according to participants’ responses and situations. At the beginning of each follow-up session, the content of the previous interview was reviewed with the participant, who was invited to comment on and confirm or correct the researcher’s understanding. The repeated interviews thus served to build rapport and to deepen and clarify each case; saturation across the dataset was assessed separately, at the cross-case level (see Section 2.5).

### 2.3. Data Analysis

This study employed a multiple-case (collective) study design (Stake, 1995, 2006). Each participant constituted one case, bounded as the individual’s life-course experience of exclusion, withdrawal, and isolation, from school years to the present, as accessed through the repeated interviews described above. A qualitative approach is well suited to addressing the gaps in existing research in that, by capturing the processual and contextual meanings of participants’ situations beyond statistical figures, it can bring to light the experiences and voices of the young people themselves, which quantitative research finds difficult to explain. The analysis proceeded in two phases: within-case analysis followed by cross-case analysis. All coding and analysis were conducted in Korean by the researcher; software-assisted coding (CAQDAS) was not used, and transcripts were segmented and coded manually in Excel (Microsoft Corp., Redmond, WA, USA) worksheets, with translation into English undertaken only at the reporting stage, after the analysis was complete. The analysis centered on the verbatim interview transcripts, supplemented by field notes and observational records; information provided by staff of the recommending organizations was limited to confirming participants’ eligibility at recruitment and was neither collected nor analyzed as research data.

The procedure was as follows. First, the verbatim transcripts were read repeatedly to obtain a holistic sense of each case, while the researcher’s emerging questions and impressions were recorded as memos. Second, sentences and phrases bearing on participants’ experiences were identified, and the raw data were organized case by case to generate meaning units, segmented around the study’s focus—the lives and social-exclusion experiences of Youth NEETs—and conceptually labeled; meaning units were then grouped and progressively conceptualized into evolving categories whose interconnections were reviewed. Coding was hybrid in logic: the social-exclusion domains provided a sensitizing orientation, while the meaning units, subcategories, and categories themselves were derived inductively from participants’ accounts and revised iteratively as cases were compared, with category structures evolving until they accommodated all cases. Third, to analyze each case’s issues and context in detail, a narrative account of each individual case was composed with attention to thick description, foregrounding the experiences distinctive to that case. Fourth, after all eight within-case analyses were complete, cross-case analysis compared the cases continuously, identifying categories, subcategories, and relations among categories that ran across the individual transcripts in relation to the research questions; commonalities were first consolidated into subcategories, which were then thematized into main categories and integrated through structural description. Finally, the phenomena and meanings that had emerged were reflected upon and integrated, connecting data and context to arrive at the essential themes answering the research questions. This process yielded 33 meaning units, 11 subcategories, and five main categories (Table 2).

For reasons of space and, above all, to protect the anonymity of a small and potentially identifiable group of participants, the individual case narratives are not reproduced in this article; the Results are organized by the cross-case categories, with within-case variation indicated where relevant.

### 2.4. Ethical Considerations

Participant recruitment and data collection proceeded after approval by the Institutional Review Board of Ewha Womans University (protocol code 165-5; date of approval: 21 October 2018). Although the study was reviewed as low risk, the interviews addressed sensitive topics, including depression, psychiatric treatment, past self-harm, statements about wanting to die, family violence, and sexual harassment. Participants were therefore informed in advance that recalling past experiences and experiences of social exclusion could cause psychological discomfort, and consent was obtained on this basis. They were also told that they could take a break, decline to answer any question, or stop the interview at any time without consequence. In addition, they were provided with information on mental health counseling services and crisis helplines, and were told that referral to counseling or other support could be arranged if needed. No participant expressed acute distress during the interviews, and no referral was requested or required.

### 2.5. Rigor and Trustworthiness

Trustworthiness was pursued through multiple complementary strategies. Credibility was supported by prolonged engagement—three interview sessions per participant conducted over approximately 15 months—and by triangulation across the interview transcripts, field notes and observational records, and related literature. Dependability and confirmability were supported by memoing throughout the analysis, by the worksheet-based analytic trail of the within-case and cross-case procedure, and by peer debriefing: the emerging codes, categories, and interpretations were reviewed in ongoing consultation with the academic advisor and two peer researchers. Transferability was addressed through thick description of participants’ backgrounds and contexts, within the limits of confidentiality owed to this small and potentially identifiable group. Sample adequacy was addressed on three grounds. First, saturation was judged across the dataset rather than within individual cases: data collection and analysis proceeded concurrently, and recruitment concluded when additional cases no longer yielded new meaning units bearing on the research questions—a judgment of saturation cross-checked in consultation with the academic advisor and two peer researchers rather than made by the researcher alone (Hennink et al., 2017). Second, in terms of information power (Malterud et al., 2016), the study’s conditions favored a smaller sample: the aim was narrow, the participants were highly specific to that aim, the analysis was grounded in an articulated conceptual framework, and dialog quality was strengthened by repeated interviews. Third, the multiple-case design itself requires a case count small enough to preserve within-case depth; eight cases fall within the range conventionally recommended for cross-case analysis (Stake, 2006).

The researcher’s position was also considered reflexively. At the time of data collection, the author was a doctoral candidate in social welfare with more than 15 years of practice experience as a social worker supporting young people, including serving as the head of a social welfare organization supporting the self-reliance of vulnerable young people; neither this organization nor any organization with which it had working ties was involved in participant recruitment. This practice background facilitated rapport and familiarity with the service context, but also carried the risk of reading participants’ accounts through a practitioner’s lens. The author entered the study with the working assumption that both individual and structural factors were likely to contribute to NEET status, and sought to examine how these factors actually operated in participants’ lives rather than to confirm either view. To limit the influence of her position on the interview dynamics, the author introduced herself to participants as a researcher with experience supporting young people, without reference to her managerial role, and sought to build a comfortable, non-hierarchical relationship. Reflective and interview memos were used to record the author’s assumptions and reactions alongside the data; interpretations from earlier sessions were checked with participants in follow-up interviews (Section 2.2); and emerging interpretations were examined in peer debriefing with the academic advisor and two peer researchers holding doctorates in social welfare with experience in both qualitative research and social work practice.

All interviews were conducted, transcribed, and analyzed in Korean; translation occurred only at the reporting stage, after the analysis was complete, so that the analytic categories were derived entirely from the original-language data. Quotations were first translated by the author and refined with the assistance of an AI-based language tool (disclosed in the Acknowledgments); every translated quotation was then verified by the author against the original Korean transcript for semantic and tonal fidelity. Culturally specific expressions (e.g., ‘kangaroo tribe’) were retained with brief explanation rather than replaced by approximate English idioms. The AI tool was used only on anonymized text at the manuscript-preparation stage—no raw data, recordings, or identifying information were processed—consistent with participants’ consent to anonymized publication of their accounts.

## 3. Results

In cross-case analysis, the commonalities and differences among the cases were examined by exploring the meanings and contexts of the social exclusion experienced by the participants. The analysis produced five main categories, 11 subcategories, and 33 meaning units (Table 2). Participants had continually experienced exclusion even before entering NEET status, had arrived at NEET status through that accumulated exclusion, and remained embedded in compounded exclusion at present. The overarching context running across all cases can be summarized as ‘being trapped in a gray zone of isolation, walled in on all sides by cumulative exclusion’. Across the categories, these accumulated experiences of exclusion were manifested psycho-emotionally in forms consistent with learned helplessness, self-stigma, and avoidant coping, as described in detail below.

### 3.1. The Shadow of Marginalization

The first main category encompasses the experiences of marginalization that participants had experienced within their family and social systems in the course of growing up, before reaching NEET status.

#### 3.1.1. A Family That Could Not Be Leaned On

For most participants, the family failed to serve as a source of psycho-emotional and material protection. Conflict arising from family disruption, economic poverty, and a lack of support systems overlapped, and the burden of caring for family members with difficulties such as depression or anorexia fell back upon the participants themselves.

*My younger sister had really severe anorexia, and then bulimia. Dealing with her is incredibly hard. In my family, everyone has something wrong with them, one way or another.*

(Participant A)

Some grew up in homes where their parents’ quarrels escalated into extreme violence, and they still carry trauma that affects their mental health to this day. Conflicts rooted in drinking and economic hardship sometimes led to verbal and physical violence, breakdowns in communication, and running away from home.

*At home my dad was violent—he beat my mom badly, there was blood every day, my mom crying and screaming, him waving a knife around. I think that’s why I became mentally unstable.*

(Participant B)

#### 3.1.2. Desolate Relationships

Many participants suffered deep wounds from school violence, collective bullying, and verbal abuse, during middle and high school, and afterwards came to fear the very act of forming relationships with others.

*When I was bullied it was so hard that I even self-harmed, but now that time has passed, it fades.*

(Participant G)

Participants perceived themselves as introverted and, weighed down by feelings of inferiority, became oversensitive in relationships or could not approach others first; maladjustment to school and dropping out severed even the friendships that had remained.

*Since I quit high school partway through, I lost contact with the friends I was close to, and when I quit university too, I stopped keeping in touch with my classmates as well.*

(Participant A)

### 3.2. Being Pushed Out into the World Unprepared

The second main category encompasses the exclusion experienced in the education and training process of transitioning from school to society. Participants faced entry into society without having prepared, during their school years, for the future—for independence and a career path.

#### 3.2.1. School Years That Passed Without Meaning

Participants moved up to higher levels of schooling without motivation or goals for advancement. It was common for them to have chosen schools favorable to quick employment because of family circumstances, or to have entered unwanted schools and departments according to their grades; even those who went to university chose their majors vaguely, without basic information, which led to indifference, poor grades, and dropping out.

*I wanted to do something creative with computers, but at university, listening to things like marketing, it was so boring that I couldn’t get interested at all.*

(Participant B)

*There was actually a school I wanted to go to, but my teacher submitted the application without even talking to me. I tried to make the best of it since I ended up attending, but it wasn’t where I wanted to be.*

(Participant G)

Participants failed to adjust to school life that had begun this way, and were left neglected—students without a student’s identity. Academic achievement was low, and a lack of basic learning skills made it difficult to keep up with classes.

*(School life was) a living hell, every single day.*

(Participant C)

Two participants could not concentrate on school, staying up all night gaming and sleeping through class. Some ultimately discontinued their studies without graduating; despite their own efforts, they chose to give up midway in the face of repeated difficulties, and later regretted it as they experienced how their lack of educational credentials blocked access even to the labor market and training opportunities.

*Before returning to school I told myself I’d try hard again, but when the same things kept repeating after I went back, I felt I just couldn’t anymore.*

(Participant E)

*I should have just filled in the attendance days and graduated. Even if I didn’t like it, I should have at least gotten the diploma.*

(Participant G)

#### 3.2.2. Failing to Prepare Properly for Employment

Even for those who completed their schooling, career exploration and preparation for the future did not take place in any substantive way. They spent their leave-of-absence periods on part-time jobs without concrete preparation—grades, certificates, language skills—and were pushed out into society with only the vague awareness that ‘after graduation, one must earn money’.

*For four years of university I was busy with the student council and drinking every day, so I never even took the TOEIC once.*

(Participant B)

*Even by graduation I had no idea. All I had was the vague notion that I should earn money and get a job; I never got my cover letter reviewed or anything like that.*

(Participant D)

Some participated in vocational training such as the Employment Success Package, but many of the courses were one-size-fits-all programs mismatched with their interests and aptitudes, and provided no real help. They dropped out midway or gave up on certification exams after completion, so that the training did not lead to skill acquisition; some, relying on the experiences of people around them, came to distrust the training system itself.

*I did learn nail art with the Tomorrow Learning Card, but there was no one my age and talking with the instructor was hard. In the end I didn’t get the certificate—I just learned and left.*

(Participant H)

*If the chance comes I’ll work, but if vocational training alone got people hired, I’d have been hired long ago. It doesn’t work anyway.*

(Participant C)

### 3.3. Treated as Expendable in an Unstable Labor Market

The third main category encompasses the exclusion experienced within the labor market. Participants drifted through simple part-time jobs without properly accumulating careers, and had negative labor market experiences in poor working environments.

#### 3.3.1. Drifting Through Simple Jobs That Never Became a Career

Rather than work requiring qualifications or preparation, participants entered the labor market through part-time and contract jobs that were easy to enter, simply ‘to get by’. Most of the work consisted of simple tasks requiring no skill or expertise: day labor, serving, kitchen assistance, delivery, warehouse management, retail sales, and telephone reception.

*I did a lot of overnight convenience store shifts. I was at an izakaya for a year as a kitchen assistant and server, and almost two years at convenience stores.*

(Participant G)

*Convenience stores, supermarket assistant work; I can’t ride a bicycle, so I delivered food on foot, and I also did warehouse organizing for accessories at Dongdaemun Market.*

(Participant C)

Their first work experiences should have been the process of beginning to build skills and careers as newcomers to society, but participants were left without proper job guidance, taking on whatever tasks were thrust upon them and shouldering excessive burdens. In that process, skills and careers failed to accumulate, and amid frequent mistakes they came to see themselves as ‘people who are bad at work’.

*Learning the work was extremely hard. It felt like being abandoned, so I just did everything—logistics, cleaning, all of it. Not having a proper position made it even harder.*

(Participant E)

#### 3.3.2. An Exhausting Hand-to-Mouth Existence

Participants were exposed mainly to poor jobs requiring physical labor. They endured high-intensity physical work and low wages—working alone in a kitchen with no air conditioning in summer, moving heavy loads—and suffered pain and anger amid unjust workplace cultures and verbal abuse.

*The busiest time for a chicken restaurant is summer, but the owner said air conditioning was a waste of money and sent all the fans out to the dining area, so there was nothing in the kitchen. The heat was a big part of why I quit.*

(Participant A)

*I suffered while working. Psychologically. Every day, all day long, I was so angry it felt like it was filling me up to here.*

(Participant B)

Their work was unstable labor—intermittent, short-term employment with no experience of regular positions. They were effectively dismissed with a ‘stop coming in’ notice without due process; even knowing it was unjust, they could not muster the courage to object, and as negative evaluations accumulated, they internalized a shrinking sense of themselves in relation to work.

*At the paint factory, they told me to stop coming because I kept making mistakes and was bad at the work. I know firing someone that way is illegal, and I wanted to get compensation or at least talk about it, but I couldn’t find the courage.*

(Participant E)

The places they worked were mostly small and poorly resourced, and their labor rights as workers went unprotected. They experienced sub-minimum wages, an inability to take leave, and working for over a year without ever receiving severance pay.

*I was the only part-timer at the chicken restaurant. Even if something came up, I couldn’t take time off. If there was a gap, I had to fill it myself.*

(Participant A)

*Even after working somewhere for over a year, I’ve never once received severance pay.*

(Participant C)

### 3.4. Failing on the High Threshold of Employment

The fourth main category shows the process by which participants, frustrated at the high threshold of employment and unable to start again, became worn out and gave up, thereby entering NEET status.

#### 3.4.1. Unable to Even Begin

Participants commonly carried a sense of being lost, unable even to set a career direction, not having found what they were good at or what they wanted to do. Without direction, they simply collided with reality and accumulated only experiences of failure.

*The really important thing is this: I don’t know what work I should do. And there’s nothing I feel confident about.*

(Participant B)

*There’s nothing I’m good at, and I don’t have a good academic background. I think that’s where I got stuck. There was nothing I could do. I didn’t even think to ask anyone, and there was no one to teach me.*

(Participant D)

In particular, they repeatedly ran up against a barrier whereby, if one’s educational credentials or certificates fall short of a certain standard, even entry into the employment system is blocked. Even those who had graduated from university and held qualifications were frustrated by the excessive competition over credentials —what Korean job seekers call “specs,” the accumulated degrees, certificates, and test scores expected of applicants.

*Real employment, not part-time work—I have neither the skills nor the education. I didn’t finish school and I don’t have certificates. I want to stop doing part-time jobs, but there’s nothing I can get hired for.*

(Participant G)

*My specs weren’t bad, so I thought I’d get a job quickly, but these days even certificates don’t really count as specs anymore.*

(Participant B)

In the end, constantly running up against the limits of age, education, and career history, they arrived at the conclusion that ‘there is nothing I can do’.

*At first there was no education requirement, so I applied, but later they notified me that I couldn’t be accepted because I lacked the credentials.*

(Participant H)

#### 3.4.2. Worn Out and Wanting to Give Up

Wanting to work, they attempted job seeking and certificate acquisition in their own ways, but as failure repeated itself from the preparation stage onward, they grew exhausted and lost confidence. Analytically, these accounts trace the erosion of perceived control that precedes helplessness: as effort and outcome became decoupled through repeated rejection, expectation itself was progressively abandoned.

*The experiences of failure just keep accumulating. I think they piled up without my even realizing it.*

(Participant B)

Even while thinking they must escape NEET status, self-distrust and fear weighed more heavily than effort, and they shrank as they compared themselves with others. Not knowing where to begin, they wandered or waited for opportunities as the gap in their histories lengthened; anticipating rejection, they lowered their sights or gave up looking toward employment altogether. Notably, however, not a single participant planned never to work in the future.

*I looked at job sites occasionally, but I couldn’t do it actively. I had no confidence that I could do anything.*

(Participant E)

*I don’t want to do part-time work, but I know I have to work. So for now I’m just waiting.*

(Participant G)

### 3.5. Being Trapped in a Gray Zone of Isolation

The fifth main category encompasses the state in which participants, living as NEETs with nowhere to turn and no one to ask for help, saw their daily lives collapse amid isolation, and the process by which this led to self-blame and, ultimately, self-exclusion.

#### 3.5.1. Nowhere to Lean On

Belonging neither to school nor to the labor market, participants were deprived of opportunities for participation in both education and work, and the absence of belonging led to exclusion from the social relationships that form in those settings. Even in joining clubs or programs, they encountered exclusion based on the ‘employed/student’ distinction.

*When my mom and my sister leave for work, I’m the only one left at home. At times like that I really feel cut off from the world. Like I don’t belong anywhere.*

(Participant B)

*I tried to join a club, but the categories were ‘employed/student’. They talked as if it was awkward for someone who doesn’t have a job to join.*

(Participant E)

Except for the two participants living in residential facilities and the one living alone, all lived with their families and depended on them largely for subsistence; but those families were themselves poor and unsupportive of the NEET life, so participants lived as members of the ‘kangaroo tribe’—a Korean term for adult children who remain financially dependent on their parents—barely leaning on family while watching their reactions.

*Both daughters are unable to work properly. Mom needs to prepare for her old age, but whatever she earns just gets eaten up. I feel suffocated, and so does she.*

(Participant B)

*I got scolded a lot, and lived on the allowance they gave me.*

(Participant F)

Beyond family, support systems for sharing feelings or obtaining information were lacking, and participants grew increasingly isolated, carrying the awareness that they themselves—who could do nothing—did not even qualify as targets of support programs.

*Honestly, I felt wronged. When my younger sister came home with the youth allowance. I’m the one doing nothing with no money—why give it to her, who’s earning?*

(Participant A)

#### 3.5.2. A Collapsed Daily Life

As NEET life stretched on, daily life collapsed. Participants tried ‘not to think at all’ in order to forget reality, and the helplessness that had begun after past wounds and failures became chronic, reaching a state in which they could no longer control their own lives.

*I don’t want to think, so I just fall into games. When I think, I get depressed and suffocated and feel like nothing will ever work out, so I just give up thinking.*

(Participant B)

An irregular life with day and night reversed became entrenched, and games and the internet became objects of immersion occupying most of daily life rather than leisure. Some spent whole days logged into closed online communities in their areas of interest, passing time in virtual space.

*There’s an anonymous community where nobody knows who anyone is and people just post about daily life. There, strangely, I felt less lonely. I practically lived there.*

(Participant D)

Experiences of seclusion—avoiding going out and staying only at home—also appeared, and with unstable psychological states, irregular living, binge eating, insomnia, and drinking overlapping, warning signs appeared in body and mind. Half the participants were receiving psychiatric treatment for depression, anxiety, obsessive symptoms, or insomnia, but were generally passive about it.

*Recently I stayed home for about a year. Going out was too hard. I have to steel myself completely, and even if there were a space for NEETs, I don’t think I could go out alone.*

(Participant A)

#### 3.5.3. Negating Oneself Through the Eyes of Others

Participants repeatedly experienced blame and pressure from society and family directed at themselves as NEETs, and internalized it, negating themselves. They watched the reactions of the family they lived with, and the pressure intensified the longer their NEET period lasted. In terms of the conceptual framework, these accounts show societal labeling being converted into self-stigma within the home—the family, as the most proximate audience, administering the NEET label daily.

*‘Stop gaming, get a job, you’re pathetic.’ The look in their eyes is different, you know. I can feel it.*

(Participant B)

Participants perceived themselves—dependent on family, unable to engage in economic activity—as problematic beings who ‘just eat up food and fail to function as a person’, and came to feel guilt and self-loathing before a reality that would not change. Together with the compulsion that they ‘must do something productive’, some revealed the fear that if things continued this way they might become hikikomori.

*I felt so sorry and so guilty and so tormented that I couldn’t even speak. Ah, I really am just eating up food—I blamed myself like that a lot.*

(Participant E)

*When you hear the word NEET, what comes to mind is, like, trash. I thought I’d be able to do everything well, but that’s me now.*

(Participant B)

This, in turn, led to self-blame, attributing the cause of arriving at NEET status entirely to oneself.

*(About not being able to get a job) Because I’m the problem.*

(Participant G)

Some expressed complex emotions in words such as ‘I want to disappear’ and ‘I want to die’. These were not actual suicide attempts, but were interpreted as expressions of frustration at a situation they could not escape by their own strength, and as desperate appeals for help—not knowing how to live.

*They say the person who says they want to die is the one who most wants to live. They want to live but don’t know how, so ‘I want to die’ is their way of asking for help instead. That’s what I think.*

(Participant D)

## 4. Discussion

### 4.1. Research Conclusions

The findings show that the Youth NEETs in this study had experienced social exclusion continuously, repeatedly, and cumulatively across multiple dimensions of the life course—family, school, the labor market, and social relationships. Exclusion surfaced as deprivation and dispossession, maladjustment and neglect, dropping out, restricted participation and opportunity, and relational rupture and isolation; rather than operating separately, these dimensions interlocked to produce further exclusion, taking on an overlapping and circular character.

The starting point of NEET status was ‘the shadow of marginalization’, arising from conflict and marginalization in family and peer relationships. The family system negatively affected participants from the formation of the self and across their entire lives, and participants were excluded from the protection, care, and preparation for adulthood they should rightfully have received. At the same time, they took on the role of compensating for the deficiencies in unstable families—while, paradoxically, the family remained the only support they could lean on. In the education and training process that followed, they were ‘pushed out into the world unprepared’. Amid low academic achievement and indifference, relational maladjustment and school violence, they experienced maladjustment and departure, were excluded from opportunities for learning, development, and social participation, and their lack of human capital in turn blocked entry into the labor market. Formal supports such as career assessments and vocational training existed, but maladjustment in a state of career immaturity instead undermined their confidence. Through this process, they came to experience learned helplessness and diminished self-efficacy.

In the labor market, participants were ‘treated as expendable in an unstable labor market’, unable to settle stably. Most had work experience and a clear will to work even now; although classified as non-job-seeking NEETs, they crossed back and forth over the boundary between NEET and non-NEET for subsistence. Yet they could not make meaning of their work experiences as ‘employment’, nor could they form a worker identity. The instability of the secondary labor market pushed them back to the margins, and without developing any real sense of the discrimination they faced in terms of labor and social rights, they came to resign themselves to, and comply with, structural limits. The result was repeated failure of entry—‘failing on the high threshold of employment’. Behind the appearance of ‘doing nothing’ lay the dream of doing ‘proper work’, but before barriers demanding credentials and competition they could not even begin; as failures repeated, fear grew, confidence drained away, and they remained in the gap.

In the place where all these exclusions had accumulated, the young people were trapped in ‘a gray zone of isolation, walled in on all sides’. Within a ‘collapsed daily life’, they engaged in avoidant coping and showed reduced behavioral activation—immersion in games and the internet, and the reversal of day and night. Experiencing psycho-emotional and physical difficulties and exclusion from relationships and support systems, they arrived—amid the deprivation of belonging, the loss of opportunities for self-realization, and depression, frustration, and isolation—at ‘self-exclusion’ through self-stigma and the formation of a negative self-concept. Their difficulties must be approached as a social problem interlocked with multidimensional social exclusion, extending beyond individual-level problems of maladjustment and failure. At the same time, these experiences of exclusion were manifested among the participants as psycho-emotional problems—learned helplessness, diminished self-efficacy, self-stigma, avoidant coping and reduced activation, and isolation—which call for active intervention and support.

Why did participants internalize failure so strongly? Three features of the Korean context converge here. First, a strongly credentialist and hyper-competitive educational and labor market culture frames employment outcomes as direct measures of personal worth, so that repeated rejection is read as evidence of personal deficiency rather than structural scarcity. Second, familist norms of co-residence and support mean that most participants remained materially dependent on families who were themselves strained—the so-called ‘kangaroo tribe’ situation—placing them under daily observation by the very people whose expectations they felt they had failed; stigma was thus administered and absorbed within the home. Third, in the absence of institutional belonging, no alternative identity (student, worker, trainee) was available to buffer the NEET label. Under these conditions, the pathway from repeated uncontrollable failure to learned helplessness, and from societal labeling to self-stigma, operates with particular force.

The findings extend the quantitative literature by supplying the experiential pathway beneath its associations. Quantitative studies report robust links between NEET status and depression (Gariépy et al., 2022) and between career expectations and withdrawal behavior (Xu et al., 2025); the present cases show how such associations are lived—how repeated labor-market rejection erodes perceived control, how family dependence converts economic strain into daily relational stigma, and how withdrawal then feeds back into deepened exclusion. While a qualitative design cannot establish causal order, it renders visible mechanisms that cross-sectional correlations cannot, and identifies textures that surveys structurally miss: families operating simultaneously as the last source of support and the most immediate source of pressure; online spaces providing escape and a residual thread of connection while entrenching withdrawal; and a sustained wish to work persisting behind the appearance of ‘doing nothing’.

Relative to the existing Korean qualitative literature, what is new here is the combination of population and process. Prior studies have synthesized the subjective world of NEET youth (S. H. Kim, 2023) or traced life-history turning points (Noh, 2021); this study focuses specifically on non-job-seeking Youth NEETs—the group most detached from institutional contact and least visible to services—and reconstructs the cumulative process by which exclusion becomes withdrawal and isolation. In doing so it shows that non-job-seeking status was not an expression of low motivation: every participant wished to work and continued to cross the labor-market boundary intermittently for subsistence. Their withdrawal is more accurately characterized as produced by ongoing exclusion, insecure living conditions, exhausted confidence, and fear of renewed rejection. The findings also resonate with accounts of NEET ‘churn’ (Baldry et al., 2019), in which advance and retreat repeat without linear progression.

The findings also position the Korean gray zone in relation to the hikikomori literature. Participants shared with hikikomori accounts the reversal of day and night, immersion in online spaces, and retreat from relationships (Baek & Yoon, 2025; Li & Wong, 2015), yet none exhibited sustained physical confinement, and all maintained intermittent, subsistence-driven contact with the labor market. The gray zone is thus conceptually distinct: a liminal, unstable state between participation and seclusion, from which movement toward either recovery or hikikomori-type withdrawal remains possible. This processual view suggests that the boundary between NEET status and seclusion is porous, and that the gray zone constitutes a critical window for intervention.

Although grounded in the Korean context, the account developed here may have relevance beyond it; any such claim, however, must be made cautiously, given that it derives from eight service-connected participants in a single metropolitan area. The study proposes a mechanism—cumulative multidimensional exclusion internalized as learned helplessness, self-stigma, and avoidant coping, and enacted as withdrawal—that linked structural conditions to individual behavior among these participants. Whether a similar pathway operates elsewhere is an empirical question; the mechanism may nonetheless offer a lens worth examining in other settings where precarious secondary labor markets, credential-based gatekeeping, and family-dependent welfare arrangements coincide, and it suggests that the boundary between NEET status and hikikomori-type seclusion may be better understood as processual than categorical. For behavioral science, the findings suggest that constructs typically measured as individual-level variables may be produced relationally and structurally, and that interventions targeting motivation alone may be insufficient where the underlying conditions of exclusion remain untouched.

### 4.2. Implications

This study raises the need for a full-fledged discussion on conceptualizing Youth NEETs and setting target criteria appropriate to the Korean context, and calls for in-depth examination of the complex and multi-layered exclusion phenomena revealed among them. It further suggests diversifying approaches to the various pathways and contexts that lead into NEET status, and shifting support for the youth transition toward an integrated perspective that is not confined to economic viewpoints and labor-market-entry-centered policies (Gunnes et al., 2025). Psycho-emotional support must be strengthened so that those who suffer psycho-emotional and everyday difficulties amid social exclusion and withdrawal can move beyond depression, frustration, and isolation to recover their lives. Furthermore, stage-based, empowerment-oriented interventions are needed for their recovery, social participation, and revitalization.

In terms of practice, personalized self-reliance support programs that integrate welfare and employment need to be developed. Support responding to the compounded difficulties of Youth NEETs and their families, expanded psycho-emotional support prior to employment, and active interventions that take into account the influence of adverse childhood experiences (ACEs) are required. In addition, communities should provide decent, protected transitional jobs in which young people can experience positive self-formation and psychosocial stability; preventive interventions such as self-exploration and career preparation within the adolescent school curriculum should be instituted; and programs should be established to support, stage by stage, those undergoing re-socialization as they move out of NEET status. Each of these directions is anchored in participants’ own accounts—the need for psycho-emotional support prior to employment follows from the finding that confidence collapsed before job seeking ceased, the need for protected transitional work opportunities from accounts of unprotected and discarded labor, and the need for family-inclusive approaches from the dual role families played—while stage-based, empowerment-oriented program models are further supported by prior research (Gunnes et al., 2025).

In terms of policy, vulnerable young people such as Youth NEETs need to be recognized not only as targets of employment policy but also as targets of social welfare services and of psychosocial empowerment, with the corresponding institutional foundations put in place.

### 4.3. Limitations and Future Research Directions

The temporal position of the data deserves explicit consideration. The interviews were conducted in 2018–2019, immediately before the COVID-19 pandemic, and this article is a delayed publication of doctoral research completed in 2020. Rather than diminishing their relevance, this timing gives the data a distinct analytic value: they document that severe withdrawal and isolation among the Korean Youth NEETs in this study predated the pandemic and arose from cumulative, structural processes of exclusion, and thus cannot be attributed to pandemic-related disruption alone. Developments since then indicate that the phenomenon has expanded rather than receded. The Korean government commissioned its first nationwide survey of socially isolated and withdrawn youth in 2023; the resulting report put the isolated youth population at approximately 540,000 as of 2021—up from about 340,000 in 2019—and secluded youth at 2.4% of those aged 19–34, some 244,000 nationally, and dedicated support programs have since been expanded (S. Kim et al., 2023; K. Kim, 2024). Because the contribution of this study is a process-oriented account of how cumulative exclusion culminates in withdrawal—rather than a point estimate of prevalence—its findings speak to the mechanisms that current policy must address, while post-pandemic changes in the scale and texture of youth isolation remain an important subject for future research.

This qualitative study of eight participants was conducted just before the COVID-19 pandemic, and it carries the limitation of a restricted sample—participants residing in a specific region and recruited through organizational recommendation. There is also a time gap between the interviews and the present. In particular, the study does not reflect the situation after the pandemic, in which NEET status has increased across society and isolation and withdrawal have deepened. Nevertheless, in that the NEET experiences presented here constitute contexts that had already formed prior to and independently of the special circumstances of the pandemic, the study remains meaningful for understanding Youth NEETs and the necessity of supporting them. Future research on Youth NEETs should be conducted in multifaceted and in-depth ways with participants of more diverse characteristics. Three further limitations deserve note. Because participants were recruited through recommendations from community organizations, the sample may over-represent young people retaining at least a minimal point of contact with services; the most severely isolated Youth NEETs—those wholly disconnected from any institution—may be under-represented, and their withdrawal may run deeper than documented here. The interviews were retrospective, so accounts of earlier school and work experiences are subject to recall limitations. Finally, the e-mail interview format, adopted as a reasonable adjustment enabling the participation of one severely withdrawn young person, affords less interactional depth and carries inherent privacy limitations relative to face-to-face interviewing. Accordingly, the findings are not intended to be generalized to Korean Youth NEETs as a whole; their transferability to other groups and settings, including other societies, should be judged in light of the contextual description provided here and examined in further research.

## Figures and Tables

**Table 1:** General characteristics of the participants.

Case	Gender	Age	NEET Duration (Cumulative)	Education	Economic Status	Residence	Family	Psycho-Emotional and Relational Difficulties
A	F	23	2 years	University dropout	Basic livelihood recipient	With family	Mother, younger sister (father deceased)	Depression
B	F	29	1 year 6 months	University graduate	Non-recipient (low income)	With family	Mother, younger sister (parents divorced)	Depression
C	M	28	3 years (5 years)	High school graduate	Basic livelihood recipient	Independent	Father (left home), younger sister (moved out)	Depression
D	F	34	1 year	University graduate	Non-recipient (average)	Residential facility	Father, mother, older brother (moved out)	Depression, interpersonal avoidance
E	M	29	1 year (3 years)	University dropout	Non-recipient (average)	Residential facility	Father, mother, younger sister	Depression, ADHD
F	M	28	5 years	High school graduate	Non-recipient (low income)	With family	Father, mother, younger brother	Interpersonal avoidance, history of game addiction
G	M	24	1 year (3 years)	High school dropout	Basic livelihood recipient	Independent	Younger sister (parents divorced, mother deceased)	-
H	F	22	3 years	High school graduate	Non-recipient (low income)	With family	Father, mother, older brother, younger brother (with disability)	Interpersonal avoidance

Note: Information in the ‘Psycho-Emotional and Relational Difficulties’ column is based on participants’ own accounts during the interviews; no clinical screening, assessment, or diagnosis was conducted by the researcher. Treatment histories are stated only where participants reported having received treatment.

**Table 2:** Main categories, subcategories, and summary of meaning units.

Main Category	Subcategory	Summary of Meaning Units
The shadow of marginalization	A family that could not be leaned on	A family in need of care; a wounded family
	Desolate relationships	Victimization by bullying and violence; difficulty forming relationships
Being pushed out into the world unprepared	School years that passed without meaning	Advancing to school without motivation or goals; neglected school life; drifting and dropping out of school
	Failing to prepare properly for employment	Not preparing for life after graduation; vocational training that did not help
Treated as expendable in an unstable labor market	Drifting through simple jobs that never became a career	Simple work requiring no qualifications; experience and skills that never accumulated
	An exhausting hand-to-mouth existence	Work that wore down body and mind; being used up briefly and discarded; unprotected labor rights
Failing on the high threshold of employment	Unable to even begin	Feeling lost; barred from entering the employment system; concluding there is nothing one can do
	Worn out and wanting to give up	Repeated failure; vanishing confidence and growing fear; wandering, waiting, and giving up
Being trapped in a gray zone of isolation	Nowhere to lean on	Belonging nowhere; a family barely leaned on; no one to turn to; deepening isolation
	A collapsed daily life	Helplessness; reversed day and night; immersion in games and the internet; seclusion; warning signs in body and mind
	Negating oneself through the eyes of others	Blame and pressure from others; ‘failing to function as a person’; self-blame; wanting to disappear

## Data Availability

The data presented in this study are not publicly available due to privacy and ethical restrictions applicable to qualitative interview data.

## Appendix A

**Table A1:** Main domains and representative questions of the semi-structured interview guide.

Domain	Representative Questions and Topics
1. Background information	Age; gender; educational attainment; religion; family composition and relationships; economic circumstances; physical and mental health; duration of NEET status; route of connection to the recommending organization; qualifications and job-related capabilities.
2. Experiences before NEET status	School: “What was school life like for you?” (satisfaction, relationships, difficulties; reasons for discontinuation where applicable). Work: “What work have you done?” (duration, tasks, working conditions, reasons for leaving). Job seeking: “Tell me about your most recent experience of looking for work. Why do you think it did not lead to employment?” Experiences of welfare services, employment services, and volunteering.
3. Current daily life	“What is your daily life like these days?” (a typical week; main activities); “Where do you mainly spend your time, and what do you do there?”; “What do you do when you are alone?”; use of computer, TV, and social media; leisure; “When something is troubling you, who do you talk to?”
4. Perceptions of NEET status	“What have you experienced living as a NEET?”; “What comes to mind when you hear the word ‘NEET’?”; “Where do you feel you stand in society?”; “What are the reasons you are not working and not in education?”; the lives of other NEETs around the participant.
5. Social exclusion domains (researcher’s sensitizing reference)	Economic, educational, employment and labor, relational, social-participation, and other exclusion experiences. Items in this domain were not posed directly to participants; they served as the researcher’s reference for probing and contextual verification, with accounts elicited through the open questions of Domains 2–4.
6. Future outlook	“How do you think about your future self? What do you imagine your life will look like a year from now?”; “What would you need for a better future?” (personal, family, community, and government/policy levels).
